# Vitamin C and immune cell function in inflammation and cancer

**DOI:** 10.1042/BST20180169

**Published:** 2018-10-09

**Authors:** Abel Ang, Juliet M. Pullar, Margaret J. Currie, Margreet C.M. Vissers

**Affiliations:** 1 Mackenzie Cancer Research Group, Department of Pathology and Biomedical Science, University of Otago, Christchurch 8011, New Zealand; 2 Centre for Free Radical Research, Department of Pathology and Biomedical Science, University of Otago, Christchurch 8011, New Zealand

**Keywords:** ascorbate, demethylation, hydroxylases, hypoxia inducible factors, vitamin C

## Abstract

Vitamin C (ascorbate) is maintained at high levels in most immune cells and can affect many aspects of the immune response. Intracellular levels generally respond to variations in plasma ascorbate availability, and a combination of inadequate intake and increased turnover during severe stress can result in low plasma ascorbate status. Intracellular ascorbate supports essential functions and, in particular, acts as an enzyme cofactor for Fe- or Cu-containing oxygenases. Newly discovered enzymes in this family regulate cell metabolism and epigenetics, and dysregulation of their activity can affect cell phenotype, growth and survival pathways, and stem cell phenotype. This brief overview details some of the recent advances in our understanding of how ascorbate availability can affect the hydroxylases controlling the hypoxic response and the DNA and histone demethylases. These processes play important roles in the regulation of the immune system, altering cell survival pathways, metabolism and functions.

## Background

Humans have an absolute requirement for vitamin C (ascorbate) as part of their diet, and deficiency due to inadequate intake is associated with a plethora of symptoms, reflecting the diverse functions attributed to the vitamin [1–4]. There is widespread belief that ascorbate supports the immune system, and claims along this line are frequently encountered, including on commercially available dietary supplements. Since its discovery more than 80 years ago, the function of ascorbate in the immune system has been the subject of much research and more than a little controversy. One of the main drivers of this interest is that leukocytes accumulate the vitamin to high intracellular concentrations, signalling an important role for it in these cells [5–9].

Additionally, in the last 15 years, the many cofactor functions of ascorbate have come to the fore. It is well established that ascorbate is a specific cofactor for many biosynthetic enzymes including dopamine β-hydroxylase, which converts dopamine to norepinephrine, and the collagen prolyl and lysyl hydroxylases which form cross-links to stabilise the tertiary structure of collagen [10–13]. More recently, however, it has become apparent that ascorbate is also a cofactor for newly characterised hydroxylases that regulate gene transcription and cell signalling pathways [14,15]. These hydroxylases belong to the family of Fe-containing 2-oxoglutarate-dependent dioxygenases; members of this family are widespread throughout biology, and include enzymes involved in biosynthesis, post-translational protein modification and the oxidative demethylation of methylcytosine and methylated histone residues [16–20]. Examples of these enzymes include *N*-trimethyllysine hydroxylase and γ-butyrobetaine dioxygenase that synthesise carnitine [16], and the prolyl, lysyl and arginine hydroxylases that modify collagen and the alpha regulatory subunit of the hypoxia-inducible factors [19,20].

This short review will focus on the cell signalling and gene regulatory (cofactor) actions of vitamin C and their potential roles in regulating the immune system. The contributions of ascorbate as an antioxidant in immune cells have been well reviewed by others [21–27] and will not be discussed here. We will consider the functional effects of ascorbate on cells of both the innate and adaptive immune responses that particularly reflect the cofactor activity of ascorbate and include a discussion of the role of vitamin C on immune cells in cancer.

## Ascorbate levels in immune cells

The optimal concentration of ascorbate required for its cofactor activity supporting the Fe- and Cu-containing enzymes *in vitro* is in the mM range [28], similar to the intracellular levels measured in many cell types [1,29,30]. The ascorbate content of immune cells is also in this range and reflects plasma availability. Intracellular ascorbate concentrations in circulating lymphocytes, monocytes and neutrophils have been reported to be ∼3.5, ∼3 and ∼1.5 mM, respectively, when plasma levels are at least 50 µM, reflecting the status in healthy individuals consuming ≥100 mg ascorbate daily [8,31]. However, when plasma levels fall below 50 µM, immune cell ascorbate content decreases, with intracellular concentrations at ∼1.5, 1.2 and 0.5 mM in lymphocytes, monocytes and neutrophils, respectively, when plasma levels are ≤20 µM [8,31]. Plasma levels below 23 µM represent a state of hypovitaminosis C and are commonly seen in individuals with low fresh fruit and vegetable intake [32–36]. In addition, there is substantial evidence that plasma and cellular ascorbate levels are depressed in conditions of active inflammation [37–40] and in cancer patients [41–43], including patients with haematological cancers [44–50]. Severely depleted plasma levels of ≤20 µM are commonly reported, particularly in very ill patient populations [37,40,44]. Ascorbate loss during illness is thought to reflect increased turnover due to oxidative and metabolic stress [51,52]. This variable availability of ascorbate may modulate ascorbate-dependent enzyme reactions and thereby affect immune cell function.

The cellular ascorbate content referred to above applies to mature circulating white blood cells. A recent report indicated that haematopoietic and multipotent stem cells and haematopoietic progenitor cells in the bone marrow contain 2- to 20-fold more ascorbate than differentiated cells and that increased ascorbate content correlated with increased expression of the specific ascorbate transporter, SVCT2 [53]. This information suggests an essential role for ascorbate in bone marrow stem cell differentiation. Evidence for this is accumulating, with recent reports of ascorbate-mediated regulation of epigenetic programming and differentiation in bone marrow stem cells and particularly in myeloid leukaemia cells containing mutations in *TET2* or *IDH1* [54,55]. For more in-depth information, the reader is referred to recent reviews of this interesting and fast-developing field of research [56,57].

## The role of ascorbate in the hypoxic response and implications for immune cell function

The hydroxylase enzymes that regulate the activity of the hypoxia-inducible factors (HIF)s require ascorbate for optimal activity [28,29]. The HIFs are controlled by hydroxylation of proline and asparagine residues on the regulatory alpha subunit and, in response to changes in oxygen availability, they direct the transcription of hundreds of genes via the hypoxia response element [58–61]. The dependence of the hydroxylases on ascorbate as a cofactor has been demonstrated in cell-free systems [28,61,62], with other reducing agents such as glutathione being very much less effective as a recycler of the hydroxylase active site Fe^2+^ [28,63–65]. Depleted intracellular ascorbate levels have been shown to contribute to the up-regulation of HIF activation, particularly under conditions of mild or moderate hypoxia [29,66].

**Figure 1.**
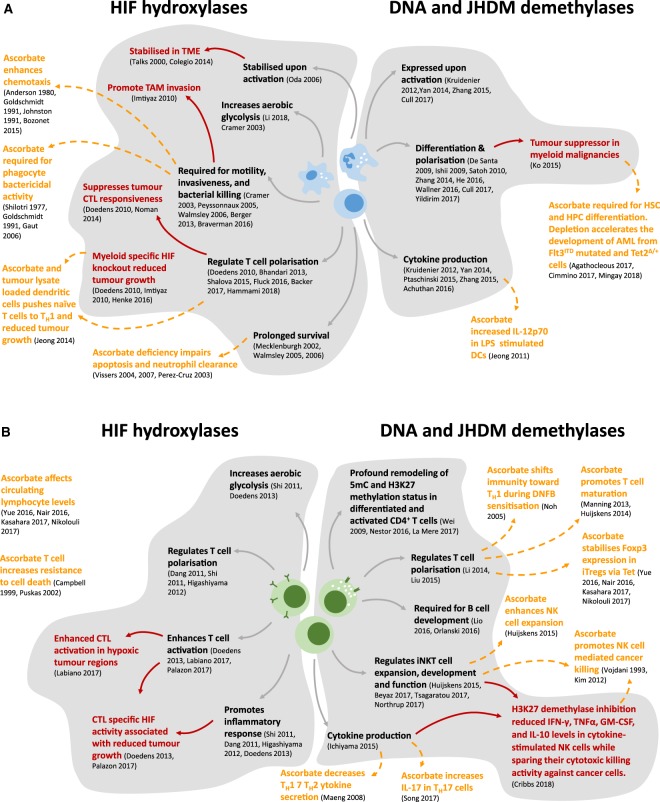
A summary of the recently reported effects of ascorbate-dependent processes in immune cells. (**A**) Effects on myeloid cells and (**B**) lymphoid cells. Effects shown in black font represent a reported role of HIF, TET or Jumonji demethylases, text in red indicates a reported effect of HIF, TET or Jumonji in the context of cancer and orange text indicates an effect of ascorbate on immune cells. The inter-relationships between these are indicated by arrows. References from the Figure: Achuthan 2016 [135]; Agathocleous 2017 [53]; Anderson 1980 [164]; Backer 2017 [88] Berger 2013 [101]; Beyaz 2017 [162]; Bhandari 2013 [165]; Bozonet 2015 [108]; Braverman 2016 [86]; Campbell 1999 [166]; Cimmino 2017 [142]; Colegio 2014 [90]; Cramer 2003 [84]; Cribbs 2018 [163]; Cull 2017 [131]; Dang 2011 [112]; De Santa 2009 [137]; Doedens 2010 [93]; [113]; Fluck 2016 [115]; Gaut 2006 [107]; Goldschmidt 1991 [106]; Hammami 2018 [116]; He 2016 [167]; Henke 2016 [94]; Higashiyama 2012 [114]; Huijskens 2014,2015 [122,123]; Ichiyama 2015 [148]; Imtiyaz 2010 [87]; Ishii 2009 [139]; Jeong 2011,2014 [95,96]; Johnston 1991 [168]; Kasahara 2017 [158]; Kim 2012 [169]; Ko 2015 [170]; Kruidenier 2012 [141]; LaMere 2017 [151]; Labiano 2017 [119]; Li 2014 [153]; Li 2018 [83]; Lio 2016 [171]; Liu 2015 [154]; Maeng 2008 [172]; Manning 2013 [124]; Mecklenburgh 2002 [98]; Mingay 2018 [55]; Nair 2016 [156]; Nestor 2016 [147]; Nikolouli 2017 [157]; Noh 2005 [173]; Noman 2014 [92]; Northrup 2017 [161]; Oda 2006 [82]; Orlanski 2016 [174]; Palazon 2017 [117]; Perez-Cruz 2003 [103]; Peyssonnaux 2005 [85]; Ptaschinksi 2015 [146]; Puskas 2002 [175]; Satoh 2010 [134]; Shalova 2015 [89]; Shi 2011 [111]; Shilotri 1977 [176]; Song 2017 [159]; Talks 2000 [177]; Tsagaratou 2017 [160]; Vissers 2004,2007 [178,179]; Vojdani 1993 [180]; Wallner 2016 [132]; Walmsley 2005,2006 [99,100]; Wei 2009 [152]; Yan 2014 [140]; Yildirim 2017 [136]; Yue 2016 [155]; Zhang 2014,2015 [133,145].

The interaction between ascorbate and the HIFs is relevant to the function of immune cells in both inflammation and cancer. Inflammatory sites are known to be under hypoxic stress, potentially as a consequence of the increased oxidative metabolism of inflammatory cells [67–69]. Growing tumours are also well characterised as being hypoxic tissues due to rapid proliferation and outgrowth of the established blood supply [70,71]. The resulting up-regulation of the HIFs is instrumental in the activation of glycolysis, angiogenesis, resistance to chemotherapy and the promotion of a stem cell phenotype, thereby promoting tumour growth and metastasis [59,72,73]. At inflammatory sites and in tumour tissue, the hypoxic environment affects immune cell function and, given the interdependence between the activation of the HIFs and cellular ascorbate [14,29,74–78], we propose that many effects of ascorbate on immune cell function are likely to reflect the regulation of HIF-mediated functions. Figure 1 shows a summary of the interactions that are discussed in the sections below.

## Effects of HIFs and ascorbate on immune cells

Immune cells undergo dramatic metabolic changes following activation, and increased aerobic glycolytic activity and fatty acid oxidation have been observed [79,80]. These metabolic changes, once thought to be a consequence of cell activation, are now being re-examined as a mechanism for phenotype switching, termed metabolic reprogramming (reviewed in ref. [81]). Central to this switch are the HIF proteins which not only up-regulate the glycolytic machinery but also direct the inflammatory and immune response (reviewed in ref. [80]).

### Monocytes/macrophages

The high ascorbate concentrations in monocytes [31] may be related to their dependency on HIF for many essential functions. HIF-1 has been shown to be activated in monocytes following activation with phorbol esters [82] and pathogenic stimuli [83–86], even under non-hypoxic conditions. That HIF activation is an integral part of monocyte function is indicated by the demonstrations that HIF-1α or HIF-2α deletion in myeloid cells caused profound impairment of cell aggregation, motility, invasiveness and bacterial killing [84–86], resulting in decreased bacterial resistance and failure to restrict systemic spread of a localised infection [85–87]. HIF-1/2 appears to be important for monocyte-mediated host defence; HIF-1 activation has been shown to contribute to disease progression in colitis and myeloid HIF-1α knockout shifts the balance to an anti-inflammatory phenotype resulting in a less severe inflammation [88]. The sepsis-related host immunosuppressive monocyte phenotype has also been shown to be mediated by chronic HIF-1α expression, resulting in supressed pro-inflammatory cytokine expression and increased ability to induce Treg cell polarisation [89].

In cancer, activation of HIF-1/2 in monocytes has been implicated in the development and phenotype of tumour-associated macrophages [87,90]. This is associated with an increased M2-like gene profile, increased expression of immunosuppressive and pro-tumour proteins such as arginase 1, iNOS and VEGF, as well as induction of PD-L1 expression [87,90–92]. These changes lead to greater monocyte/macrophage tumour invasion [87] and tumour cytotoxic T-cell suppression [92,93]. Interestingly, a macrophage-targeted HIF-1α and HIF-2α knockout resulted in delayed tumour progression in models of breast tumour, fibrosarcoma and colitis-associated colon carcinoma [87,93,94].

The potential complexity of ascorbate engagement with immune cells in the tumour microenvironment is well demonstrated by the observations that dendritic cells treated with ascorbate secreted increased levels of IL-12p70 after activation with LPS and induced more Th1 cytokine and IFN-γ, but less Th2-cytokine, IL-5 expression in naive T cells [95]. Ascorbate-treated dendritic cells also increased the frequency of IFN-γ + T cells when co-cultured with both CD4^+^ and CD8^+^ T cells and demonstrated an improved anti-tumour effect [96].

### Neutrophils

Neutrophils are short-lived cells that are the first responders to an inflammatory challenge. Their recruitment to, and clearance from, inflammatory sites is dependent on the regulation of cell death and survival pathways [97]. It appears that HIF-1 and ascorbate are intimately involved in determining neutrophil cell fate. Hypoxia has been shown to prolong neutrophil survival via activation of HIF-1 and its downstream pathways [98–100]. HIF-1 activation also enhanced overall neutrophil antibacterial function as demonstrated by increased susceptibility to bacterial keratitis in mice when HIF-1 was inhibited [101]. This was supported by findings of delayed rates of apoptosis and enhanced bacterial phagocytosis under normoxic conditions in neutrophils from patients with a monoallelic mutation of von Hippel Lindau protein who exhibit a ‘partial hypoxic’ phenotype [99]. These results suggest that a functional hypoxic response supports neutrophil function at hypoxic inflammatory sites *in vivo*. A similar anti-apoptotic phenotype in ascorbate-deficient neutrophils was shown to be associated with HIF-1 activation under normoxic conditions [102]. Recognition of aged neutrophils by macrophages was also reported and neutrophil clearance from an inflammatory site was delayed in deficient cells [102]. Interestingly, increasing neutrophil ascorbate content was found to inhibit neutrophil Fas-induced cell death [103] as well as the rates of neutrophil and monocyte apoptosis in patients with sepsis [104]. Also, in the ascorbate-dependent Gulo^−/−^ mouse, a high ascorbate diet was found to increase circulating granulocyte and monocyte numbers [105].

Not all effects of ascorbate on neutrophil function will be HIF-related. Severe ascorbate deficiency has been shown to impair neutrophil bactericidal ability towards phagocytosed pathogens following infection with actimoycetes and K pneumoniae [106,107], possibly as a result of altered oxidative capacity. Neutrophils from individuals with suboptimal circulating ascorbate levels showed a modest increase in neutrophil chemotaxis and oxidative burst *ex vivo* following supplementation to restore vitamin C status to healthy levels [108]. Vitamin C deficiency also increased the generation of neutrophil extracellular traps (NETs) in the Gulo^−/−^ mouse [109].

### T cells

Differentiation of CD4 T cells dictates the type of inflammatory response occurring via the development of different T-helpers and iTreg subsets and their corresponding effector function [80,110]. Therefore, depending on the nature of the insult or source of inflammation, the prevailing ratio and species of T cells could alter the outcome. HIF-1 appears to play an important, although unresolved, role in T-cell differentiation. For example, HIF-1α T-cell-targeted knockout protected mice from autoimmune neuro-inflammation and was associated with a shift from T_H_17 to Treg response, possibly by increasing glycolysis [111–113], while the opposite was observed in irritable bowel disease where T-cell HIF-1α knockout increased T_H_1 and T_H_17 leading to severe colonic inflammation [114]. HIF-1α-mediated myeloid- and dendritic cell-driven differentiation of T cells also greatly affected the inflammatory outcome; HIF-1α knockout in myeloid cells resulted in lesser T_H_17 prevalence and decreased inflammation [88]. In dendritic cells, HIF-1 knockout resulted in impaired Treg development and increased inflammation [115], and HIF-1-mediated events were reported to limit Th1 cell development by preventing IL-12 production and to exacerbate *Leishmania* infections [116].

Apart from T-cell differentiation, HIF-1 has also been shown to affect T-cell activation and function. HIF activation enhanced the expression of effector molecules, co-stimulatory receptors, activation and inhibitory receptors, and key transcriptional regulators of effector and memory cell differentiation [113,117]. However, this was in contrast with a previous report showing higher levels of pro-inflammatory cytokines, stronger antibacterial effects and much better survival of septic mice with T-cell targeted deletion of HIF-1α [118].

In cancer, HIF-1 activation is associated with expression of CD69 (a marker of activated T cells) on cytotoxic T lymphocytes (CTLs) in hypoxic regions of tumour, suggesting a pro-tumour killing role for HIF-1α [119]. This is supported by two studies, showing that T-cell HIF-1 activation significantly delayed tumour growth [113] and, conversely, accelerated tumour progression in the presence of HIF-1α knockout CTLs [117] in a murine model of ectopic B16 melanoma.

There have been many studies that have suggested that ascorbate influences lymphocyte differentiation, including early studies that indicated that increased circulating lymphocytes were associated with ascorbate availability [120,121]. High ascorbate supplementation for one-year also significantly increased all circulating leukocytes, including lymphocytes, in the SMP30KO ascorbate-dependent mice [105]. Ascorbate was required for the progression of mouse bone marrow-derived progenitor cells into functional T-lymphocytes and also increased the NK cell population *in vitro* [122–124]. Many of these effects show a significant correlation with the regulation of the TET and Jumonji demethylases and epigenetic changes, rather than with the expression of HIF-1. This topic is discussed in the following section.

## Ascorbate and the regulation of epigenetics in immune cells

In mammals, one of the most widespread epigenetic modifications is DNA cytosine methylation which can be actively reversed by the TET enzymes that catalyse the oxidation of 5-methylcytosine (5mC) to 5-hydroxymethylcytosine (5hmC), 5-formylcytosine (5fC) and 5-carboxylcytosine (5caC) [125]. Ascorbate availability enhances TET activity [126,127] through its cofactor function, likely maintaining the active site Fe^2+^ of these dioxygenases [128]. Although other reducing agents could reduce Fe^3+^ and promote TET activity in a cell free system, ascorbate was shown to be the most efficient [128] and glutathione was incapable of increasing murine embryonic TET activity compared with equimolar ascorbate [126,127]. The Jumonji C domain-containing histone demethylases (JHDMs) are also members of the Fe- and 2-oxoglutarate-dependent dioxygenase family and similarly to TETs, full enzyme activity of JHDMs occurs in the presence of ascorbate [129,130]. The JHDMs are the third and largest class of demethylase enzymes, capable of removing all three histone lysine-methylation states through oxidative reactions [130].

### Monoytes/macrophages

Epigenetic regulation plays an important role in macrophage differentiation, with rapid TET-dependent demethylation observed in colony-stimulating factor-1-differentiated human monocytes [131,132]. TET2 transcription was further induced by LPS but not by IL-4 stimulation [131]. The genes affected by TET-mediated demethylation are part of ten consolidated pathways related to the regulation of actin cytoskeleton, phagocytosis and the innate immune system [132], and in macrophages, TET2 is thought to restrain the inflammatory response by up-regulating expression of genes involved in dampening Toll-like receptor 4 signalling [131]. This notion is supported by a report showing TET2 represses IL-6 production during LPS-induced inflammation and that TET2 knockout exacerbates the expression of macrophage pro-inflammatory molecules such as IL-6, MCP-1 and MCP-3 in response to LPS stimulation, resulting in an enhanced inflammatory response [133].

The JHDM enzyme JMJD3 is expressed in monocytes/macrophages and is inducible by differentiating factors [134–136] as well as by pathogenic [137,138] and damage-associated molecules [139,140]. Although JMJD3 has been shown to affect gene expression in macrophages, the role of JMJD3 in macrophage function is still unclear. For example, 70% of macrophage–LPS–inducible genes were found to be JMJD3 targets but only a few hundred genes, including inducible inflammatory genes, were moderately affected by JMJD3 deletion [137]. However, Kruidenier *et al*. [141] demonstrated a drastic drop in LPS-induced cytokine expression using a specific JMJD3 inhibitor and siRNA, among them TNF-α. In contrast, Satoh et al. showed no effect on M1 cytokine secretion following LPS stimulation in JMJD3 knockout macrophages including TNF-α [134]. Contradictions aside, two studies looking at macrophage response to parasitic infection have associated JMJD3 demethylation activity with acquisition of an M2 phenotype, demonstrated by up-regulation of M2 proteins such as Arg1, Ym1, Fizz1, MR and iNOS [134,139]. Two other studies have associated JMJD3 activity with an M1 macrophage phenotype following serum amyloid A stimulation [140] and in arthritis [135] resulting in induction of pro-inflammatory cytokines.

Epigenetic processes regulated by the demethylases are associated with leukaemogenesis and ascorbate availability has been closely linked to this phenomenon. As mentioned above, haematopoietic stem and progenitor cells (HSPCs) accumulate high intracellular concentrations of ascorbate, and this is essential for HSPC differentiation via support of TET2 activity [53]. TET2 inhibition in HSPCs by ascorbate depletion retards differentiation and increases HPSC frequency. *TET2* mutations are also known to co-operate with FLT3^ITD^ mutations to cause acute myeloid leukaemia [53]. Ascorbate depletion coupled with FLT3^ITD^ mutations was adequate for leukaemogenesis [53]. It appears then, that ascorbate accumulation within HSCs promotes TET function *in vivo*, limiting HSPC frequency and suppressing leukaemogenesis. These findings were corroborated in part by another group that described the use of ascorbate as a combination therapy for treating leukaemia [142]. Patients with leukaemia often have low plasma ascorbate levels [44,47–50] and the capacity for ascorbate to influence the epigenetic drivers of some leukaemias has led to conjecture that increased ascorbate supply may provide clinical benefit to some individuals with leukaemia. Two recent publications have provided support for this hypothesis [143,144].

### Dendritic cells

DNA demethylation changes occur during the development of monocytes into immature DCs and mature DCs [145]. TET2 represses late-phase expression of dendritic cell pro-inflammatory molecules such as IL-6, MCP-1 and MCP-3 in response to LPS stimulation and TET2 knockout results in a greater degree of inflammatory response in mice challenged with LPS and colitis [133]. KDM5B acts to repress type I IFN and other innate cytokines in DCs to promote an altered immune response following RSV infection that contributes to the development of chronic disease [146].

### T cells

Widespread DNA methylation remodelling has been reported at genes and cell-specific enhancers with known T-cell function during human CD4^+^ T differentiation [147,148], and TET2 was reported to be the critical DNA demethylase involved in the differentiation of T_H_1 and T_H_17 cells, leading to activation of effector cytokine gene expression [148]. TET2 has also been shown to regulate CD8^+^ T-cell fate, particularly in formation of memory CD8^+^ T cells [149]. Prolonged antigen stimulation in peptide immunotherapy is associated with demethylation of conserved regions of PD-1 promoter, possibly via TET, leading to sustained PD-1 expression in CD4^+^ effector T cells [150].

Profound demethylation of histone H3K27 is observed after activation in CD4^+^ T cells and corresponds to pathways crucial to T-cell function, including T-cell activation and the regulation of the JAK/STAT pathways [151,152]. Deletion of the histone demethylase JMJD3 was found to regulate gene expression resulting in T_H_2 and T_H_17 differentiation and inhibiting T_H_1 and Treg cell differentiation via altered methylation status of H3K27 and/or H3K4 [153,154].

Recent studies focusing on the role of ascorbate in T-cell differentiation and function suggest close alignment with epigenetic regulation and demethylase activity. Initial work showed ascorbate to be required for the progression of mouse bone marrow-derived progenitor cells into functional T-lymphocytes *in vitro* and *in vivo* by a JMJC-mediated process [123,124]. Subsequent studies reported ascorbate-mediated stabilisation of *Foxp3* expression in TGF-β-induced Tregs by TET enzymes [155,156]. Also, ascorbate enhanced alloantigen-induced Treg suppressive capacity in skin allograft and GVHD in mice was attributed to the stabilisation of *Foxp3* expression, presumably via demethylation of *Foxp3* and other Treg-specific epigenetic genes [157,158]. Apart from Tregs, ascorbate has also been implicated in the maintenance of T_H_17 phenotype by increasing IL-17 expression in T_H_17-differentiated T cells via reduced trimethylation of histone H3 lysine 9 (H3K9me3) in the regulatory elements of the IL-17 locus [159].

### NK cells

Many recent studies have demonstrated the impact of TET- and JHDM-mediated demethylation on NKT cell development, proliferation and function [160–162]. Interestingly, inhibition of the H3K27 demethylase reduced IFN-γ, TNF-α, GM-CSF and IL-10 levels in cytokine-stimulated NK cells while sparing their cytotoxic killing activity against cancer cells [163].

## Summary

The demonstrated dependency of the Fe-containing 2-oxoglutarate-dependent dioxygenase family on ascorbate availability and the involvement of members of this family of enzymes on many immune cell functions provide a rational basis for the belief that ascorbate supports the immune system. Ascorbate availability will influence HIF activation and immune cell function in hypoxic inflammatory and tumour environments, affecting the resolution of inflammation and potentially tumour survival in as yet unknown ways. There is also an impressive amount of information emerging that highlights the impact of the TET DNA demethylases and some histone demethylases on epigenetic remodelling of immune cells. These enzymes have also been shown to be highly responsive to ascorbate, and new insights into ascorbate function in immunity will no doubt continue to emerge.

## Abbreviations

CTLs: cytotoxic T lymphocytes
HIFs: hypoxia-inducible factors
HSPCs: haematopoietic stem and progenitor cells
JHDMs: Jumonji C domain-containing histone demethylases
NETs: neutrophil extracellular traps
